# Supplementary material for: Kmt2c/Mll3 Haploinsufficiency Causes Autism-like Behavioral Deficits in Mice
Source: Biomolecules. 2025 Nov 4;15(11):1547. doi: 10.3390/biom15111547 (PMC12650575; doi:10.3390/biom15111547)
Supplement: Supplementary file 1 [file biomolecules-15-01547-s001.zip › biomolecules-3927307-supplementary.pdf]

Supplementary Methods

Open field test

Animals were placed in an apparatus (L: 67.7 cm; W: 50.8 cm; H: 50.8 cm) to move freely for 10 min. The total distance traveled and the amount of time the animal spent in the center (29 cm × 20 cm) were counted by Ethovision XT tracking software (Noldus, Leesburg, VA).

Elevated plus maze (EPM) test

Mice were placed in the center of a plus maze that was elevated 50 cm above the floor with two opposite open arms and two opposite closed arms (each arm was 88 cm long, and 28 cm-height walls only on the closed arms) arranged at right angles. The number of entries and time spent in the closed and open arms were monitored for 10 min. The behaviors were video recorded and automatically scored using Ethovision XT tracking software (Noldus, Leesburg, VA).

Social preference test

An apparatus (L: 101.6 cm; W: 50.8 cm; H: 50.8 cm) containing three chambers with retractable doorways allowing for access to side chambers was used to perform a three-chamber social interaction assay. Animals were habituated to the apparatus for 1 day before testing. During the habituation, two empty capsules (inverted pencil cup, D: 10.2 cm, H: 10.5 cm) were placed in the corner of the chambers, and an upright cup was placed on top of each capsule to prevent the subject mouse from climbing on top. Animals were allowed to explore all three chambers of the apparatus for 10 min. The test was composed of two phases with different stimuli in each of the side chambers. The phase 1 contained two identical nonsocial stimuli (folded papers), and the phase 2 contained a nonsocial (NS) stimulus (a woodblock) and a social (Soc) stimulus (an age-, sex-, strain-matched mouse). Each stimulus was placed inside a capsule placed in the corner of the chamber. The test animal was placed in the center chamber and was free to explore the apparatus for 10 min in each phase, while it was returned to its home cage during the 10-min intervals between phases. The chamber was cleaned with 75% ethanol after each phase. Interaction time was counted based on the "investigating" behaviors of the test animal to each stimulus. Computer running Ethovision XT tracking software (Noldus, Leesburg, VA) measured the time of the test animal spent at the proximity of the capsule (distance of animal head to cup edge: ≤3.5 cm). The preference index was calculated as: [time spent on social stimulus (Soc) − time spent on non-social stimulus (NS)]/ [total time exploring the social and non-social stimuli (Soc + NS)] x 100%.

Self-grooming

Mice were scored for spontaneous grooming behaviors when placed individually in a clean cage. The cage was lined with a thin layer of bedding (~1 cm) to reduce neophobia but prevent digging, a potentially competing behavior. Prior to the testing period, animals were allowed to habituate to the novel environment for 10 min. Each mouse was rated manually for 10 min on cumulative time spent grooming.

Barnes maze test

Mice were placed on a round platform with eight equally spaced holes at the edge, one of which was attached with an escape box (correct hole). Bright overhead light was applied as a weak aversive stimulation to increase the motivation to escape from the circular platform. During the two learning phases (5-min interval) (information acquisition), mice were allowed to explore the platform using distal visual cues until finding the correct hole and entering the escape box. Then, mice were placed in their home cage to rest for 15 min. In the memory phase (information retention and retrieval), the escape box was removed, and mice were put back on the platform to explore for 5 min. Mice spent the time with their noses oriented toward the hole within 3.5 cm of the hole edge was considered. The time spent on the correct hole (T1) and the other seven incorrect holes (T2) were

counted. Spatial memory index was calculated by T1/T2. The behaviors were video recorded and automatically scored using Ethovision XT tracking software (Noldus, Leesburg, VA).

Novel object recognition test

The test was composed of three phases: habituation (no objects), familiarization (two identical objects "familiar-A," 5 min), and test [(familiar-A) and a new, different object ("novel-B"), 5 min] separated by a short delay period (5 min). The room was illuminated by indirect white light. All objects were made of plastic toys (height, about 5 cm) with similar textures, colors, and sizes but distinctive shapes. The mouse was removed from the arena and placed in its holding cage at each interval between phases. During habituation, mice were placed into the open field apparatus (L: 67.7 cm, W: 50.8 cm, H: 50.8 cm) for 5 min. During familiarization, two identical objects were placed in the opposite corners counterbalanced, and the animals were allowed to explore the objects for 5 min. During test phase, mice were placed in the same apparatus, one object of the pair was replaced with a novel object, and they were allowed to freely explore for 5 min. Mice spent the time with their nose oriented toward the object within 3.5 cm of the object's edge, and/or touching it with the nose was considered, but not sitting on the object. Total exploration time of the familiar and novel objects was recorded. The discrimination index was calculated as: [time spent on novel object (B) − time spent on familiar object (A)]/[total time exploring both objects (B + A)] for the test session. The behaviors were video recorded and automatically scored using Ethovision XT tracking software (Noldus, Leesburg, VA).

Rotarod test

To assess motor coordination and balance, an accelerating rotarod (San Diego Instruments, San Diego CA) was used. Mice were placed on a cylinder, which slowly accelerated from 4 to 40 r.p.m. over a 5-min test session. The task requires mice to walk forward to remain on top of the rotating cylinder rod. The latency to fall was automatically shown by the machine.
